# The Phenotype of Monocytes in Anterior Uveitis Depends on the HLA-B27 Status

**DOI:** 10.3389/fimmu.2018.01773

**Published:** 2018-07-30

**Authors:** Maren Kasper, Karoline Walscheid, Björn Laffer, Dirk Bauer, Martin Busch, Lena Wildschütz, Bo Wang, Karin Loser, Thomas Vogl, Rafael S. Grajewski, Thomas Langmann, Arnd Heiligenhaus

**Affiliations:** ^1^Department of Ophthalmology and Ophtha-Laboratory at St. Franziskus Hospital, Münster, Germany; ^2^University of Duisburg-Essen, Essen, Germany; ^3^Department of Dermatology, University of Münster, Münster, Germany; ^4^Institute of Immunology at University of Münster, Münster, Germany; ^5^Department of Ophthalmology, University of Cologne, Cologne, Germany; ^6^Chair of Experimental Immunology of the Eye, Department of Ophthalmology, University of Cologne, Cologne, Germany

**Keywords:** immunopathogenesis, monocytes, uveitis, HLA-B27, autoimmunity, cytokine, myeloid-derived suppressor cells

## Abstract

HLA-B27 is the allele most frequently associated with human anterior uveitis. The majority of HLA-B27-positive [acute anterior uveitis (AAU)] patients develop clinically distinct symptoms with acute symptomatic onset of flare and a recurrent disease course characterized by a massive cellular ocular infiltrate during uveitis relapse. By contrast, uveitis in HLA-B27-negative [idiopathic anterior uveitis (IAU)] patients tends to develop a clinically less fulminant, more chronic, and typically asymptomatic disease course. To analyze systemic immune responses in the different uveitis entities, we analyzed peripheral blood cells by flow cytometry. In addition, as a pro-inflammatory biomarker serum, S100A8/A9 levels were quantified by ELISA from patients with AAU (*n* = 27) and IAU (*n* = 21), and in healthy controls (*n* = 30). Data were obtained either during active uveitis flare or after 3 months of inactivity. IAU patients showed a transiently increased frequency of CD56- and CD163-positive monocytes and of both granulocytic myeloid-derived suppressor cells and Th17 cells during active uveitis. By contrast, AAU patients showed an elevated frequency of monocytes, activated T cells, and elevated S100A8/A9 serum levels during clinically quiescent disease. The differentially regulated response of both innate and adaptive immune cells in the blood may be related to the clinically distinct characteristics of the two different uveitis entities.

## Introduction

Uveitis describes an inflammatory phenotype within the eye globe that is classified into various anatomical subgroups (1). Anterior uveitis (AU) is defined as inflammation confined to the anterior eye segment, involving the iris and ciliary body. Non-infectious, acute anterior uveitis (AAU) associated with presence of the HLA-B27 allele is a well-defined entity and is one of the most common types of uveitis (2–5). AAU is characterized by sudden onset, with typical clinical features including pain, eye redness, photophobia, and blurred vision. Characteristic signs of severe uveitis episodes include cellular infiltration and fibrin formation in the anterior chamber (AC) and spill-over of infiltrating cells into the anterior vitreous. Posterior synechiae, ocular hypotony, and macular or optic disk edema are commonly noted. The episode typically heals within 4–12 weeks. Treatment of inflammation is mandatory for achieving inactivity, to prevent vision-threatening complications and severe burden of illness. In addition to topical or systemic corticosteroids, treatment modalities nowadays include disease-modifying anti-rheumatic drugs, such as synthetic, conventional, and/or biological drugs for steroid sparing.

HLA-B27 is a common risk factor for developing AAU, as reflected by approximately 40–70% of AAU patients being HLA-B27 positive in contrast to only 8–10% of the healthy population (6, 7). The immunopathogenesis of HLA-B27-associated AAU is not entirely known. AAU is commonly associated with HLA-B27-associated systemic immune-mediated diseases, e.g., reactive arthritis, spondyloarthritis, ankylosing spondylitis (AS), or inflammatory bowel disease (8, 9). Despite the significant association between HLA-B27 and autoimmune diseases, the great majority of persons carrying the HLA-B27 allele are healthy, with only about 1% developing AAU (10), thus implicating the importance of environmental factors in addition to a genetic predisposition. AAU is characterized by recurrent episodes of active uveitis. Environmental factors contributing to the occurrence of relapses have not been identified so far, but important evidence has accumulated regarding the role of bacterial triggers (11, 12). Therefore, the role of innate immune cells as monocytes is of special interest. Phenotypic analysis of circulating monocytes in patients with juvenile idiopathic arthritis-associated uveitis (JIAU) or non-infectious uveitis already showed that monocytes were involved in the course of the disease (13–15). Monocytes are part of the innate immune system and support the development of an efficient adaptive immune response. Furthermore, they also play important roles in maintaining tissue homeostasis and repair (16, 17). These multiple effector functions are related to the heterogeneity and plasticity of the different subsets of monocytic cell populations (18, 19). With increasing knowledge about their diversity and various effector mechanisms, monocytes are being placed in the focus of immunological research in infection and autoimmunity (20–23).

The goal of this study was to characterize circulating monocyte populations from patients with HLA-B27-associated AAU and HLA-B27-negative patients with idiopathic anterior uveitis (IAU), each group without associated systemic diseases, in comparison with healthy controls (HCs). A better understanding of the function and differentiation of the monocytic subpopulations during autoimmune AU may facilitate a more specific therapeutic regimen (e.g., with biologics) for these particular diseases, which are major causes of visual impairment.

## Materials and Methods

### Patients

All uveitis patients were recruited at the Department of Ophthalmology at St. Franziskus Hospital, Münster, Germany. HC individuals (matched for gender and age) were recruited among the employees of the hospital. The study protocol was approved by the local ethics committee. The study followed the tenets of the Declaration of Helsinki. Written informed consent was obtained from all patients and controls before study entry. A venous blood sample was taken from all patients and controls.

Inclusion criteria (all must be fulfilled) were patients with clinically non-granulomatous AU according to SUN guidelines (1), without a history of systemic immune-mediated disease (e.g., AS and ulcerative colitis), and aged 18–75 years. Standard laboratory parameters were tested in all patients: differential blood count, liver/kidney function tests, CRP, angiotensin-converting enzyme, soluble interleukin 2-receptor, serological testing for treponema pallidum, and an interferon-γ (IFN-γ) release assay to exclude tuberculosis. In addition, patients underwent chest X-ray and consultation with a specialist for internal medicine or rheumatology. Patients were classified as HLA-B27-negative idiopathic or HLA-B27-positive uveitis if none of the tests produced any further findings indicating associated systemic disease. Patients with a clinical appearance of infectious (e.g., HSV- or VZV-induced) uveitis or uveitis syndromes (e.g., Fuchs uveitis syndrome) were not included in the study. All uveitis patients and HCs underwent genetic testing for HLA-B27. The following study groups were included:

(1) Patients with HLA-B27-associated AAU with typical clinical signs of AAU and without inflammatory/immune-mediated systemic disease associated with uveitis. Patients presenting with active uveitis (*n* = 11) or quiescent disease for ≥3 months (*n* = 16) were included in the study.
(2) Patients with IAU with typical clinical signs of chronic-relapsing AU with insidious onset and HLA-B27 negative genotype. No inflammatory/immune-mediated systemic disease associated with uveitis was present. Patients presenting with active uveitis (*n* = 6) or quiescent disease for ≥3 months (*n* = 13) were included in the study.
(3) Age-matched healthy volunteers were asked to participate in the study. A questionnaire was used for obtaining the medical history, asking for the presence of any type of eye disease, local or systemic immune-mediated disease, any active infectious, allergic or inflammatory disease (e.g., rhinitis, fever, or common cold) or malignancy, and current anti-inflammatory systemic medication. Only healthy volunteers (HLA-B27-pos. *n* = 7, HLA-B27-neg. *n* = 23) without any infectious, inflammatory, or chronic diseases were included in the study.

### Ophthalmic Examinations

A standardized ophthalmic database was applied for the analysis. Ophthalmic observations on uveitis were documented according to the SUN criteria (1). In patients with bilateral uveitis disease, observations from the eyes with the higher AC cell grade at study inclusion were chosen for analysis.

Best-corrected visual acuity testing (in LogMAR), slit-lamp examination, Goldmann tonometry, and funduscopy were performed by two independent observers. The presence of ≥1+ cells in the AC was defined as active uveitis. Inactivity of uveitis was defined as <0.5+ cells in the AC. Vitreous haze was estimated (1).

### Test for HLA-B27 Status of Healthy Volunteers

HLA-B27 status of healthy volunteers was determined by using FACS analysis and PCR. Peripheral venous blood (5 ml) was collected in EDTA tubes (Sarstedt, Nümbrecht, Germany) from each healthy volunteer, and 200 µl of blood samples was prescreened for HLA-B27 positivity by flow cytometry using anti-HLA-B7/B27-PE (REA176) and REA control antibodies (S)-PE (Miltenyi Biotech, Bergisch Gladbach, Germany). The remaining blood volume was stored at −80°C until further analysis. Samples that were identified as being HLA-B7/-B27 positive by flow cytometry were additionally analyzed using PCR (MVZ for laboratory medicine, Koblenz, Germany) to confirm HLA-B27-positive controls. Blood samples with negative results according to flow cytometry (FACS) analysis were defined as HLA-B27-negative controls.

### Samples

Peripheral venous blood was collected from each study subject. For FACS analysis, 10 ml of blood was collected in lithium–heparin tubes (Sarstedt, Nümbrecht, Germany) and was processed immediately. Serum samples were centrifuged at 800 × *g* for 10 min immediately after acquisition, and aliquots were stored at −80°C until analysis.

### Antibodies and Reagents

For FACS staining, we used the following antibodies to target: CD16 (FITC; eBioCB16), CD14 (PE-Cy7; 61D3), CD15 (APC; MMA), IL-4 (APC; 8D4-8), and IFN-γ (4S.B3)—all purchased from eBioscience (San Diego, CA, USA)—and CD11b (FITC; ICRF44), CD56 (PE; HCD56), CD33 (PE; WM53), IL-10 (PE; JES3-9D7) CD45 (APC; HI30), CD163 (APC; GH1/61), CD3 (PE-Cy7; HIT3a), CD4 (FITC; OKT4), and IL-17A (PE; BL168)—all purchased from BioLegend (San Diego, CA, USA).

### FACS Staining and Analysis Protocol

For phenotyping, 200 µl of whole blood was stained for 30 min at room temperature (RT) with a combination of anti-human antibodies (CD14, CD16, CD56, CD11b, CD33, CD15, CD163, CD3, and CD4) according to the manufacturer’s instructions. Afterward, erythrocytes were lysed using RBC Lysis Buffer (Multi-species 10×; eBioscience) for 10 min at RT. Cells were washed twice with phosphate-buffered saline (PBS) and analyzed.

For analysis of intracellular cytokines in T cells, whole blood was diluted 1:2 with RPMI 1640 (Biochrom, Berlin, Germany) and stimulated with cell stimulation cocktail [PMA/ionomycin with protein transport inhibitors (500×); eBioscience, USA] for 6 h at 37°C. Afterward, erythrocytes were lysed using RBC Lysis Buffer (Multi-species; eBioscience) for 10 min at RT. Cells were washed twice with PBS. Cells were extracellularly stained with anti-human CD4 for 30 min at RT and washed with PBS. Cells were then fixed (IC fixation buffer; eBioscience), permeabilized (permeabilization buffer, eBioscience), and incubated with one of the following anti-human antibodies targeting IL-4, IL-10, IL-17A, and IFN-γ at 4°C for 45 min. Cells were then washed twice with permeabilization buffer and analyzed. Samples were measured using FACSCalibur (BD Biosciences) equipment and FACS Cell-Quest pro software. Data were analyzed with DakoCytomation Summit software V3.1 (DakoCytomation, Fort Collins, CO, USA). Percentage of positive cells was documented.

### Enzyme-Linked Immunosorbent Assay (ELISA)

S100A8/A9 concentrations from serum samples were determined by using a double-sandwich ELISA system as previously described in Ref. (24).

### Data Analysis

Statistical significance of patient characteristics and clinical data were analyzed by Mann–Whitney *U*-test, Chi-squared test, and Fisher’s exact test, as appropriate. Normal distribution of data was tested by Kolmogorov–Smirnov test. Flow cytometry data were analyzed by using Kruskal–Wallis and Mann–Whitney *U*-test. For statistical analysis, we used SPSS PASW Statistics version 20.0 (SPSS Inc., Chicago, IL, USA) and MedCalc (V10, MedCalc Software bvba, Belgium). *p*-Values < 0.05 were considered statistically significant.

## Results

### Patient Groups

Clinical data are summarized in Table 1. Both the HLA-B27-associated AAU and HLA-B27-negative IAU patient groups included some ANA-positive individuals (22 or 9.5%, respectively), while rheumatoid factor (RF) positivity was only detected in the AAU group (11%). Patients from both groups showed a similar occurrence of uveitis complications and had received both systemic and topical medication at similar frequencies. At study inclusion, uveitis was active in 40% of AAU patients and in 32% of IAU patients. Age at uveitis onset and at study inclusion did not differ significantly between patient groups. Subjects in the HC group were of similar age at study inclusion as patients and included an equal ratio of female and male subjects (1:1). HCs were not tested for ANA or RF. With regard to the clinical data, the patient groups were therefore homogeneously composed. Compared with the HC groups, there was a (not significant) predominance of female subjects in both patient groups, with age being comparable.

**Table 1:** Clinical data.

	Acute anterior uveitis	Idiopathic anterior uveitis	Healthy controls	*p*-Value
Patients (*n*)	27	21	30	
Male gender (*n*/%)	8 (33)	7 (29)	15 (50)	0.25^a^
HLA-B27 positive (*n*/%)	27 (100)	0 (0)	8 (26.6)	
ANA positive (*n*/%)	6 (22)	2 (9.5)	n.d.	
RF positive (*n*/%)	3 (11)	0 (0)	n.d.	
Age (mean ± SD)	37.7 ± 15.1	39.1 ± 17.3	35.5 ± 8.4	0.78^b^
Active uveitis (*n*/%)	11 (40)	6 (32)	n.a.	0.54^c^
Systemic anti-inflammatory medication (*n*/%)	10 (37.0)	7 (33.3)	n.a.	1.0^c^
Topical steroids (*n*/%)	11 (40.7)	7 (33.3)	n.a.	0.77^c^
Uveitis complications (*n*/%)	19 (70.4)	14 (66.7)	n.a.	1.0^c^
Previous ocular surgery (*n*/%)	8 (29.6)	7 (33.3)	n.a.	0.76^c^
Age at uveitis diagnosis (mean ± SD)	31.6 ± 13.0	33.9 ± 15.7	n.a.	0.55^d^

*n.a., not applicable; n.d., not determined; RF, rheumatoid factor*.

*^a^Chi-squared*.

*^b^Kruskal–Wallis*.

*^c^Fisher’s exact test*.

*^d^Mann–Whitney*.

### Phenotyping of Peripheral Blood Cells

#### Monocytes

**Figure 1:**
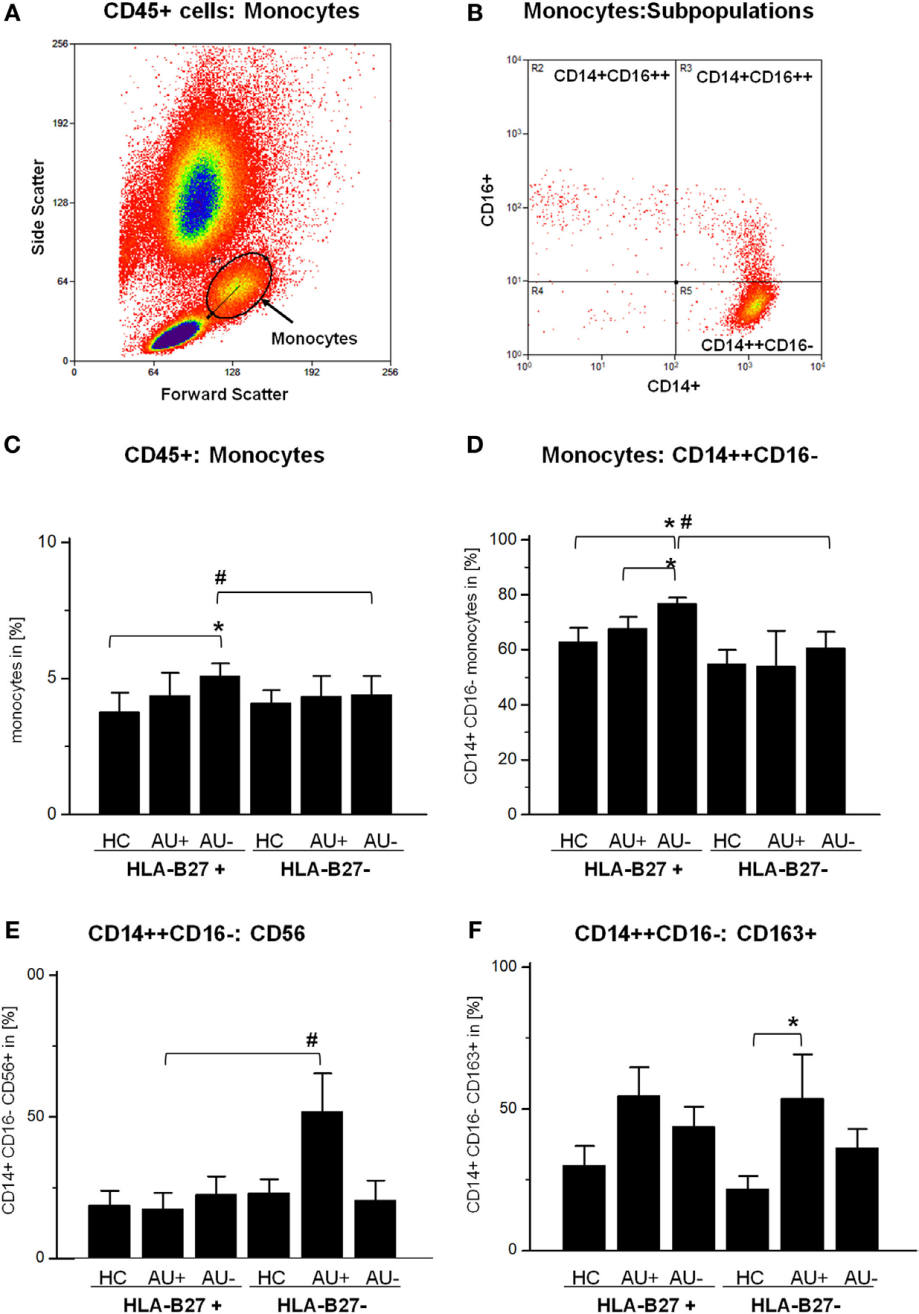
Flow cytometry analysis of CD45+ whole blood cells: **(A)** identification of monocytes in forward and side scatter plot (FSC/SSC) and **(B)** further differentiation by CD14 and CD16 expression into classical (CD14++ CD16−), intermediate (CD14++ CD16+), and non-classical (CD14+ CD16++) monocytes. Frequency of **(C)** monocytes (FSC/SSC), **(D)** CD14++ CD16− classical monocytes, **(E)** CD14+ CD16−:CD56+ monocytes, and **(F)** CD14+ CD16−:CD163+ monocytes in peripheral blood of HLA-B27-positive/-negative healthy controls (HCs), and patients with active (AU+) and inactive (AU−) anterior uveitis (*p < 0.05; Kruskal–Wallis test; ^#^*p* < 0.05; Mann–Whitney *U*-test).

The flow cytometric analysis of the CD45+ monocytic cell population in HLA-B27-positive/-negative controls and patient samples with active or inactive AAU (HLA-B27-positive) or IAU (HLA-B27-negative) showed a stable frequency of peripheral blood monocytes, while the frequency in AAU patients increased during clinical uveitis inactivity (Figures 1A,C). The monocytic population consists of three main subgroups, which can be distinguished by the expression of CD14 and CD16 (Figure 1B): CD14++ CD16− (classical monocytes), CD14++ CD16+ (intermediate monocytes), and CD14+ CD16++ (non-classical monocytes). The frequency of classical monocytes was significantly higher in AAU patients during uveitis inactivity than in both the HC and the active AAU group and than in the inactive uveitis group of the IAU cohort, too (Figure 1D). By contrast, frequencies of intermediate and non-classical monocytes remained unmodulated within all analyzed cohorts (data not shown). To further assess the phenotype and activation status of classical monocytes in AAU and IAU patients during active and inactive disease, we assessed the expression of surface marker and intracellular cytokines. Comparison of classical monocytes between HC and uveitis patients revealed that common monocytic markers, e.g., CCR2, CX3CR1, HLA-DR, HLA-ABC, IL-6, IL-1β, and TNF-α were not affected (data not shown). AAU patients showed no significantly different expression of analyzed markers during active or inactive uveitis. Within the IAU group, classical monocytes showed increased CD56 (Figure 1E) and CD163 expression (Figure 1F) during uveitis activity.

#### T Cells

**Figure 2:**
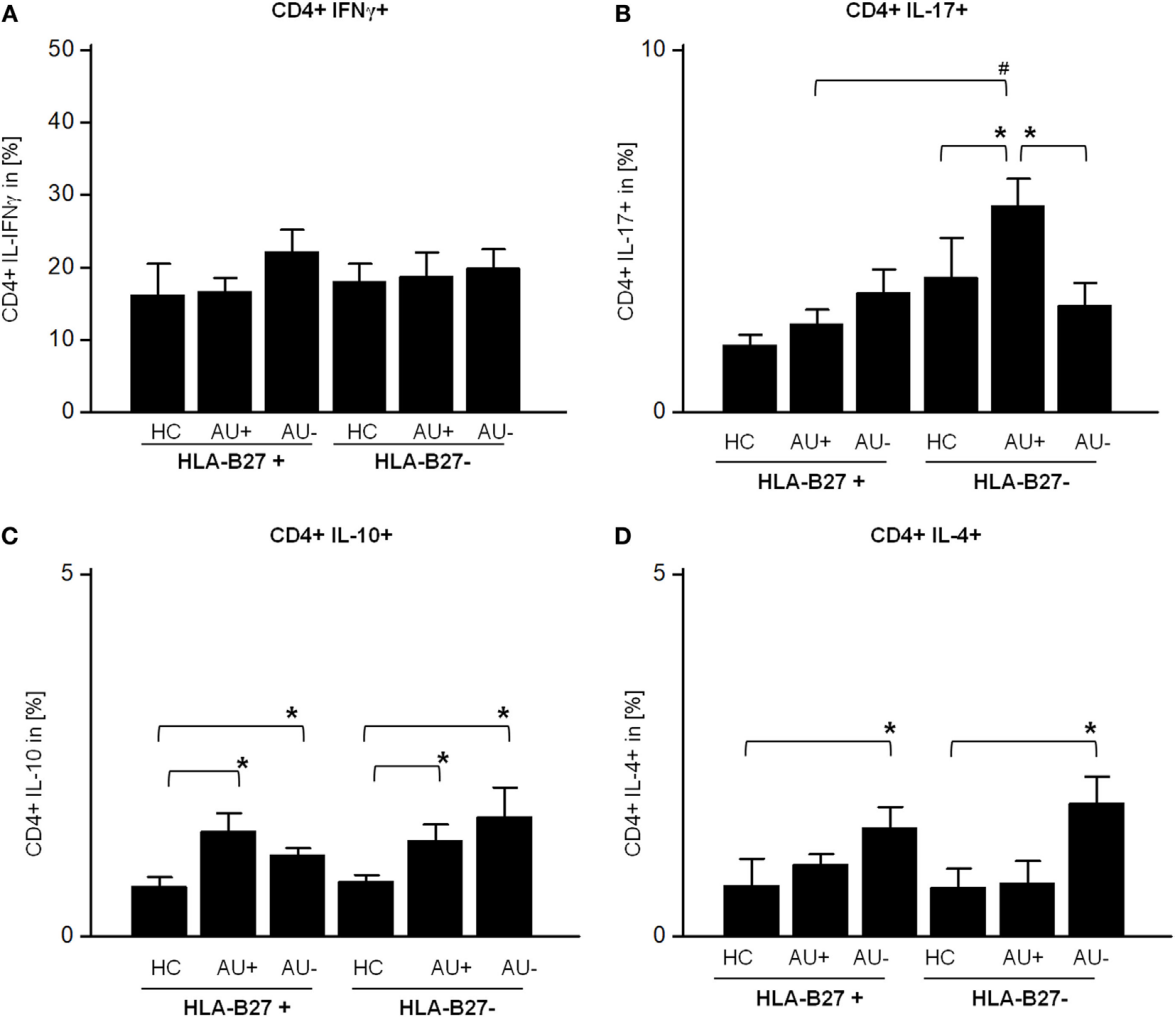
Frequency of **(A)** CD4+ IFNγ+, **(B)** CD4+ IL-17A+, **(C)** CD4+ IL-10+, and **(D)** CD4+ IL-4+ T-cells in peripheral blood of HLA-B27-positive/-negative healthy controls (HCs), and patients with active (AU+) and inactive (AU−) anterior uveitis analyzed by flow cytometry (*p < 0.05; Kruskal–Wallis test; ^#^*p* < 0.05; Mann–Whitney *U*-test).

Since monocytes influence the T-cell response and CD4+ T cells are known modulators of autoimmune uveitis in mice and humans, we analyzed their T-cell cytokine pattern next. The flow cytometric analysis of AAU and IAU patient samples with active or inactive uveitis and HC showed an unaltered frequency of peripheral CD3+ CD4+ T cells (data not shown). As previously shown for monocytes (Figures 1C,D), AAU patients displayed an increased frequency of IFN-γ and IL-17 expressing CD4+ T cells during uveitis inactivity, although the results did not reach significance (Figures 2A,B). Furthermore, AAU patients displayed an elevated frequency of IL-10 and IL-4 expressing T cells during uveitis inactivity (Figures 2C,D).

In contrast to AAU patients, the frequency of CD4+ IL-17+ T cells was significantly enhanced in IAU patients during active uveitis (Figure 2B) while the expression of IFN-γ by CD4+ T cells was not altered in IAU patients (Figure 2A). Similar to AAU patients, IAU patients displayed an elevated frequency of CD4+ IL-10+ T cells in comparison with HC, regardless of uveitis activity, and increased IL-4 expression during uveitis inactivity (Figures 2C,D).

#### Myeloid-Derived Suppressor Cells (MDSC)

**Figure 3:**
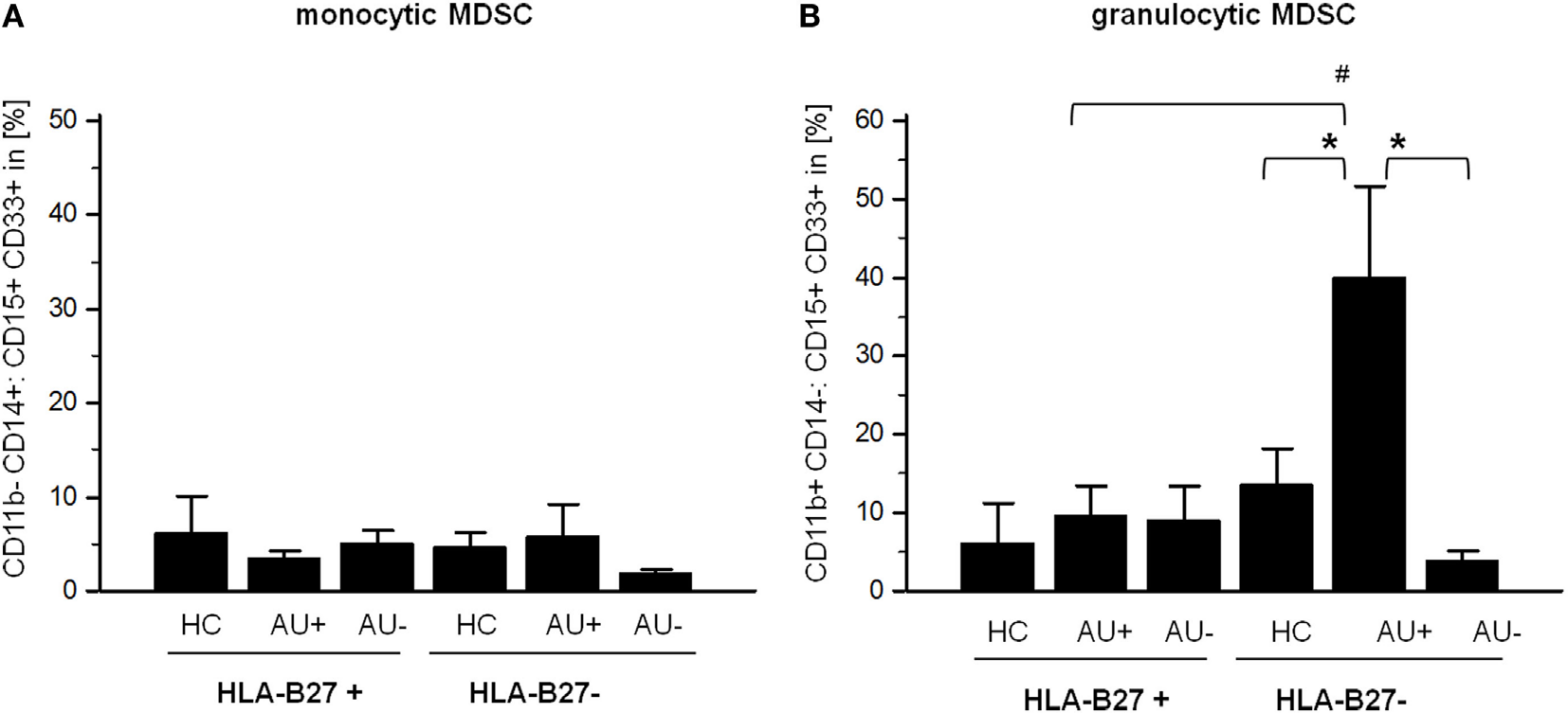
Frequency of **(A)** monocytic myeloid-derived suppressor cells (MDSC) CD11b+ CD14+:CD15+ CD33+ and **(B)** granulocytic CD11b+ CD14−:CD15+ CD33+ MDSC in peripheral blood of HLA-B27-positive/-negative healthy controls (HCs), and patients with active (AU+) and inactive (AU−) anterior uveitis analyzed by flow cytometry (*p < 0.05; Kruskal–Wallis test; ^#^*p* < 0.05; Mann–Whitney *U*-test).

Since MDSC have been implicated in regulating T-cell responses, we next analyzed the frequency of MDSC, monocytic CD11b+ CD14+ CD33+ CD15+, and granulocytic CD11b+ CD14− CD33+ CD15+ MDSC in AAU and IAU patients. As observed for Th17 cells, increased frequency of granulocytic MDSC in IAU patients with active uveitis was detected while no modulation was found in AAU patients (Figure 3).

#### S100A8/A9 Level in Sera

**Figure 4:**
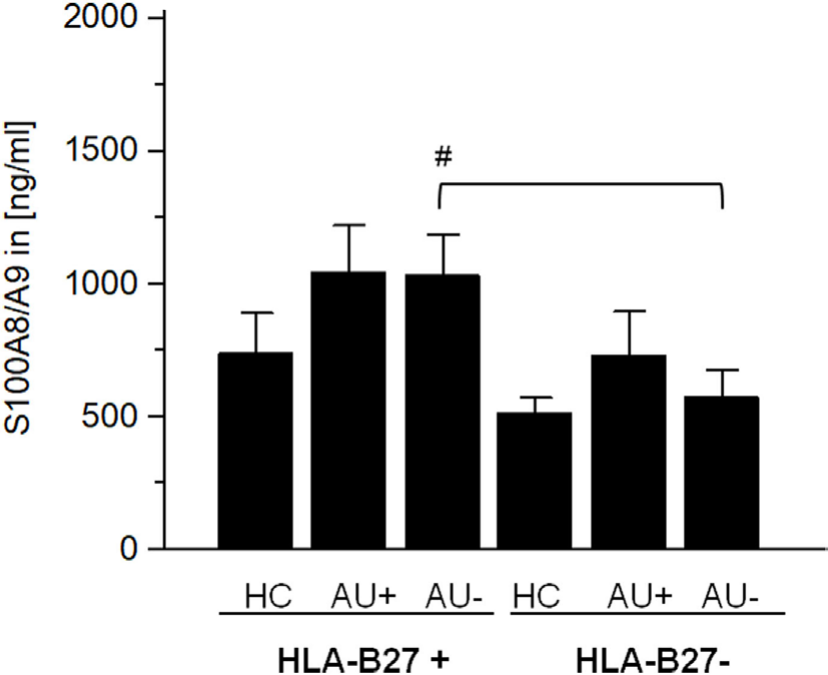
Serum level of S100A8/A9 (in ng/ml) as mean ± SEM of HLA-B27-positive/-negative healthy controls (HCs), and patients with active (AU+) and inactive (AU−) anterior uveitis analyzed by ELISA (*p < 0.05; Kruskal–Wallis test; ^#^*p* < 0.05; Mann–Whitney *U*-test).

The alarmin S100A8/A9 expressed by granulocytes and monocytes is released by activated phagocytes and as an endogenous TLR4 agonist promote inflammation during infection, autoimmunity, and cancer (25–29). Elevated S100A8/A9 serum levels serves as a sensitive biomarker of disease activity in different autoimmune diseases (e.g., rheumatoid arthritis, type I diabetes, and psoriasis) (29). As S100A8/A9 has been shown to promote the migration and infiltration of inflammatory cells into the eye during AAU and as S100A8/A9 serum levels are known to be increased in patients with AAU (30), IAU, and JIAU (31), we compared the S100A8/A9 levels in AAU and IAU patients. In IAU patients, uveitis activity was accompanied by an increase in S100A8/A9 levels, which did not reach the level of statistical significance (Figure 4). S100A8/A9 serum levels in AAU patients remained at the elevated level independently of the uveitis activity. Compared with the IAU patients, the S100A8/A9 level was significantly higher in the AAU cohort during uveitis inactivity.

## Discussion

Recently, monocytes and their diverse populations have received increased attention in autoimmune disease research (32–34). An understanding of their plasticity and phenotype in relation to disease course may promote the development of novel treatment strategies for patients with different autoimmune diseases. As their role in human uveitis is as yet largely undefined, the goal of this study was to characterize the phenotype of circulating monocyte populations from two well-defined uveitis entities, comparing patients with HLA-B27-positive AAU and HLA-B27-negative IAU, both without typically associated systemic immune-mediated diseases (e.g., AS).

Circulating monocytes represent 4–5% of the peripheral blood cells in humans (35) and are subclassified according to their CD14 and CD16 expression into three populations with functional differences, differentiating classical (CD14++ CD16−), intermediate (CD14++ CD16+), and non-classical (CD14+ CD16++) monocytes (34, 36). In this study, the absolute frequencies of monocytes were within normal ranges in all of our study groups. However, a comparison between active and inactive disease stages exhibited a higher frequency of classical monocytes in the inactive AAU cohort. Previous studies showed changes in the relative proportions of intermediate and non-classical monocytes in the context of autoimmune diseases, such as rheumatoid arthritis or multiple sclerosis, and they suggested that analyzing monocytes has potentially important implications for monitoring disease progression (23, 37, 38). In this study, no significant variations were observed during the clinical course of two distinct uveitic entities. This could be related to the absence of an associated systemic autoimmune disease in the patient cohorts. Anti-inflammatory therapy may also influence the plasticity of monocytic subpopulations, as shown previously for Crohn’s disease and non-infectious uveitis (13, 39).

With respect to phenotypic changes, no alterations were observed in monocytes of AAU patients during the course of disease, regardless of whether uveitis was active or inactive. However, elevated S100A8/A9 serum levels during uveitis activity and inactivity in AAU confirms the findings of Wang et al. who reported elevated S100A8/A9 serum levels in AAU patients in comparison with HC, independently of the uveitis activity (30). S100A8/A9 functions in general as a pro-inflammatory protein complex aggravating the disease outcome in various different diseases (29). Hereby the heterodimer acts only locally at sites of inflammation (40) and quantification of systemic inactive tetramers correlate very well with disease activity in the patient (29). Elevated S100A8/A9 levels and their association to disease activity have already been reported in other non-infectious uveitis entities, e.g., IAU and JIAU (31). The presence of these pro-inflammatory factors and the increased frequency of monocytes after more than 3 months of clinically inactive uveitis indicate a persistent, active immune status in AAU patients who had clinically recovered entirely from the previous flare. Although the elevated frequency of IFN-γ and IL-17-positive T cells in the same subgroup of patients does not reach statistical significance, these observations might point toward a persistently activated immune response even during clinical quiescence of uveitis. Previous observations are in line with our findings and report raised immune responsiveness in AAU patients, with the presence of microbial-specific antibodies showing a significant correlation to recurrent (>10 relapses) uveitis in AAU patients (11). Moreover, the elevated immune responsiveness reflected by an elevated CRP level and TNF-α release has also been shown for AU patients regardless of their HLA-B27 status (12). A similar observation of active immunity during clinically inactive uveitis has been shown previously in the aqueous humor (AH) of patients with JIAU (41). It would be of interest to determine whether the monocytic phenotype in HLA-B27-positive AAU patients with associated systemic disease, in particular spondyloarthritis, differs from the current results.

While monocytes of AAU patients were phenotypically stable during uveitis activity, IAU patients showed increased frequencies of CD56+ monocytes during uveitis activity. Indeed, such an increase in CD56+ monocytes is also known for patients suffering from Crohn’s disease or rheumatoid arthritis (42, 43). This monocytic population in particular is characterized by elevated expression of TNF-α, IL-10, and IL-23 (43) as well as an increased exertion of effector functions toward T cells (44).

Furthermore, CD163+ monocytes were increased in IAU patients during uveitis activity. The hemoglobin and high mobility group box 1 scavenger receptor CD163 is expressed by M2 monocytes/macrophages, which exacerbates their anti-inflammatory properties by clearing damaging substrates and producing anti-inflammatory mediators (45, 46). While the M1-related cytokine IFN-γ promotes apoptosis of CD163+ M2 macrophages, IL-17 promotes their survival *via* anti-apoptotic stimuli to maintain the phagocytotic capacity of M2 macrophages (47). Therefore, the increased frequency of IL-17+ T cells during active uveitis observed in IAU patients might contribute to the elevation of CD163+ monocytes in this cohort.

Myeloid-derived suppressor cells represent a subset of innate immune cells generated in the inflammatory environment, with the ability to suppress T-cell responses (48, 49). Elevated frequencies of granulocytic MDSC have been described during infection in cystic fibrosis patients, with those cells exerting a regulatory effector function against Th17 T-cell responses (50). Accordingly, in this study, elevated frequencies of granulocytic CD11b+ CD14− CD15+ CD33+ (MDSC) (51) were found in IAU patients during uveitis activity and might be linked to the increased presence of Th17 cells.

The increased frequency of Th17 cells in this study is in line with cytokine analysis of AH, wherein a correlation of increased IL-17 levels with IAU and IFN-γ levels with AAU patients has been shown, indicating a Th1-driven immune response in AAU, in contrast to a Th17-driven immunity in IAU patients (52).

In addition to the aforementioned differences, AAU and IAU patients share similarities as seen for the elevated frequency of IL-10-expressing T cells (regardless of the uveitis activity) and IL-4 expressing T cells during uveitis inactivity. We assume that this reflects the simultaneous occurrence of both pro-inflammatory and counter-regulating immune mechanisms in patients with AU.

Taken together, the elevated frequency of CD56+ and CD163+ monocytes, granulocytic MDSC, and Th17 cells during uveitis activity represents unique features in IAU patients, suggesting a Th17-driven immune response in comparison with HLA-B27-associated AAU patients. By contrast, AAU patients showed an elevated frequency of monocytes, activated T cells, and elevated S100A8/A9 serum levels even during clinically inactive uveitic disease. These findings suggest that divergent immune mechanisms may be contributing to the different clinical presentations in patients with uveitis.

## Ethics Statement

The study design complies with the standards put forth by the Declaration of Helsinki. The study was approved by the local ethics committee. All subjects provided written informed consent for peripheral blood collection.

## Author Contributions

MK, KW, DB, MB, RG, TL, and AH designed the study and wrote the manuscript. MK, BL, DB, MB, LW, BW, KL, and TV performed the experiments and analyzed the data. KW and AH collected the peripheral blood samples and provided clinical data.

## Conflict of Interest Statement

The authors declare that the research was conducted in the absence of any commercial or financial relationships that could be construed as a potential conflict of interest.

## Abbreviations

AAU, acute anterior uveitis; IAU, idiopathic anterior uveitis; MDSC, myeloid-derived suppressor cells.
